# A multi-target tracking method for UAV monitoring wildlife in Qinghai

**DOI:** 10.1371/journal.pone.0317286

**Published:** 2025-04-11

**Authors:** Guoqing Zhang, Wei Luo, Quanqin Shao, Guohong Li, Xia Zhu, Yongxiang Zhao, Dongliang Wang, Jiandong Liu

**Affiliations:** 1 North China Institute of Aerospace Engineering, Langfang, China; 2 Key Laboratory of Land Surface Pattern and Simulation, Institute of Geographic Sciences and Natural Resources Research, Chinese Academy of Sciences, Beijing, China; 3 Aerospace Remote Sensing Information Processing and Application Collaborative Innovation Center of Hebei Province, Langfang, China; 4 National Joint Engineering Research Center of Space Remote Sensing Information Application Technology, Langfang, China; 5 University of Chinese Academy of Sciences, Beijing, China; Prince Mohammad Bin Fahd University, SAUDI ARABIA

## Abstract

The Procapra przewalskii, plays a vital role in sustaining the ecological balance within its habitat, yet it faces significant threats from environmental degradation and illegal poaching activities. In response to this urgent conservation need, this article proposes a multi-object tracking (MOT) method for unmanned aerial vehicle (UAV). Initially, the approach utilizes a modified YOLOv7 network, which incorporates Group-Selective Convolution (GSConv) in its Neck component, effectively enhancing the network’s ability to preserve detailed information while simultaneously reducing the computational load. Subsequently, the Content-Aware ReAssembly of Features (CARAFE), an innovative feature upscaling method, replaces the conventional nearest neighbor interpolation to minimize the loss of critical feature data during image processing. In the tracking phase, the Deep SORT algorithm is expanded with a proprietary UAV camera motion compensation (CMC) module that eliminates the impact of UAV camera jitters. Moreover, the system has incorporated a confidence optimization strategy (COS) that enhances the tracking performance especially when the individuals are partially or fully obscured. The method has been tested on Procapra przewalskii and shown to be effective. The results show the gains in tracking metrics where the method achieved improvements of 7.0% in MOTA, 3.7% in MOTP, and 5.8% in IDF1 score compared to the traditional Deep SORT model. Improved tracking methods can alleviate the impact of occlusion and rapid movement of UAV on tracking, thereby more accurately monitoring the status of each Procapra przewalskii and protecting it. Also, the efficiency in the multi-target tracking achieved through the use of this system is sufficient for the operational demands of UAV-based wildlife monitoring, thus being a reliable tool in wildlife conservation where accurate and efficient wildlife tracking is desired.

## 1. Introduction

The Procapra przewalskii, a herbivorous mammal, primarily consumes plant matter and was historically widespread across regions including Inner Mongolia, Ningxia, Qinghai, Gansu, Xinjiang, Xizang, and the eastern sector of the Naqu region in China. Currently, its range is largely restricted to the vicinity of Qinghai Lake, with the densest populations found in the Halgai River basin, Gangcha County. This species is particularly suited to the arid, cold plateau and desert grasslands typical of high-altitude environments. It thrives in open grasslands and alpine meadows, which provide ample food resources, and prefers concealed terrains to evade predators. Previously on the brink of extinction, the Procapra przewalskii is one of the few mammals adept at surviving in such challenging conditions, playing a crucial role in sustaining the structural integrity and population equilibrium of its ecosystem. However, the gazelle continues to face threats from climate change, human encroachment, and disease spread. Despite existing legal protections, illegal poaching persists, driven by the demand for the animal’s valuable fur. This poaching pressure has significantly contributed to population declines. Additionally, the harsh environmental conditions of their high-altitude habitats—characterized by low temperatures, reduced oxygen levels, and steep terrains—complicate monitoring and conservation efforts. Given these challenges, there is an urgent need to enhance protective and monitoring strategies for the species, particularly tailored to the severe conditions of their natural environments. Effective conservation measures are vital for ensuring the long-term survival and stability of the Procapra przewalskii across its native range [1,2]. Enhancing these measures is essential for advancing conservation goals and safeguarding this key species.

In the field of wildlife conservation, various methods are utilized for monitoring species, including camera traps, remote sensing technologies, and UAV. The advantages and disadvantages of these methods are detailed in Table 1. Camera traps are particularly valuable in ecological research, as evidenced by references [3–7]. These devices capture intricate behavioral patterns and can operate over extended periods without interruption. For example, camera traps have been used to precisely monitor and assess the population densities of four distinct deer species in different regions of Australia. However, the static nature of camera traps restricts their coverage area. Additionally, maintaining these devices and retrieving data from them can prove challenging in harsh environmental conditions. On the other hand, remote sensing provides a non-intrusive method that spans large areas and does not disturb the natural behaviors of wildlife. This technology is crucial for mapping the spatial distribution and observing temporal changes in wildlife habitats. For instance, helicopters equipped with remote sensing tools have been employed to survey the composition of wildlife populations [8]. A significant drawback of remote sensing, however, is the often limited spatial resolution of the images, which may hinder detailed analysis.

**Table 1:** Comparison of advantages and disadvantages of wildlife monitoring methods.

Wildlife monitoring methods	Advantage	Disadvantage
Camera trap	Long term continuous recording, detailed capture of animal behavior data	The monitoring range is limited, and equipment maintenance and data extraction are difficult
Remote sensing	Non invasive, with a wide coverage area	Low spatial resolution, expensive equipment, complex operation.
UAV	High flexibility, wide coverage, high resolution, low invasiveness	Limited by battery life and weather conditions

The rapid evolution of UAV technology has significantly decreased operational costs, fostering its widespread integration into wildlife monitoring. UAV offer superior flexibility and enhanced image resolution compared to traditional monitoring methods such as camera traps and remote sensing, greatly improving their efficacy. These aerial platforms are extensively utilized for counting, detecting, and identifying various species within diverse ecosystems. For instance, UAV have been effectively used to survey and document population numbers of geese [9], sheep [10], and cattle [11]. Ekelbom et al. [12] reported that UAV-based imaging is particularly advantageous in regions with sparse vegetation, such as grasslands, where detection rates for large species like elephants and giraffes can reach up to 90%. Despite these advances, UAV technology continues to develop, with ongoing enhancements aimed at increasing its efficiency. The refinement of automated detection systems is a key focus area, where the integration of UAV imaging with computer vision technology proves to be the most effective strategy for the autonomous monitoring of wildlife.

In recent years, the advancement of Multi-Object Tracking (MOT) technology [13] has led to the widespread use of UAV-based MOT systems in animal monitoring. MOT typically employs two paradigms: Tracking by Detection (TBD) [14] and Joint Detection and Tracking (JDT) [15,16]. The TBD paradigm is favored for its modularity and flexibility, separating detection and tracking into distinct modules for independent optimization, making it adaptable to complex backgrounds and high-density scenes. In contrast, JDT excels in joint optimization and spatiotemporal information utilization, improving tracking continuity and stability. However, JDT is more complex, computationally demanding, and less flexible due to its tight integration of detection and tracking. As a result, the TBD approach, with its modular and adaptable nature, has become the mainstream solution for MOT systems.

The TBD framework operates on a stage-wise approach, where the initial stage is dedicated to the detection of objects, followed by the tracking of these objects using specific algorithms such as SORT [17], Deep SORT [18], ByteTrack [19], and BoT-SORT [20]. This method is generally more accurate and faster than conventional methods, particularly when employing high-performance detection and tracking systems. However, a notable limitation of the TBD framework is its practice of excluding frames with low detection scores to minimize processing time and facilitate smooth transitions between frames. This approach can result in potential tracking gaps, with missed detections and disruptions that pose significant challenges in complex environments such as wildlife monitoring. Recent studies have highlighted that although deep learning shows considerable potential in managing MOT tasks, there remains a critical need for enhancements in utility, stability, and precision, especially in densely populated and intricate scenes.

This study presents an innovative detection and tracking methodology for the Procapra przewalskii, employing YOLOv7 and Deep SORT algorithms to significantly enhance the robustness of multi-target tracking systems. The contributions of this research are detailed as follows:

1. To accommodate the limited computational resources of UAV platforms, the model incorporates lightweight convolution GSConv. This adaptation reduces the model size while maintaining optimal detection performance.
2. The introduction of the lightweight operator CARAFE during the up-sampling process replaces traditional nearest neighbor interpolation methods. This change minimizes feature information loss, ensures more complete data preservation, and enhances detection performance for Procapra przewalskii in high-altitude environments.
3. To tackle tracking failures caused by rapid horizontal, vertical, and rotational movements of UAV cameras in high-altitude areas, a Camera Motion Compensation (CMC) module has been developed. This module corrects errors in Kalman filter (KF) predictions caused by camera movement, resulting in more accurate tracking of Procapra przewalskii.
4. To address tracking failures due to occlusion at high altitudes, a Confidence Optimization Strategy (COS) has been implemented. This strategy adjusts the matching thresholds based on varying confidence levels of detection frames, mitigating the effects of occlusion and enhancing the detection accuracy and overall efficiency of the MOT system.

The structure of this article is organized into six chapters: Section 2 introduces the relevant work and background of the research; Section 3 provides an overview of the research area and the objects involved, along with a comprehensive description of the system’s overall architecture and research methodology; Section 4 details the experiments conducted and the results obtained; Section 5 offers an in-depth discussion on the findings; and finally, Section 6 summarizes the conclusions drawn from this study.

### 1.1. Relate work

Deep learning object detection networks are predominantly categorized into two types. The first type encompasses two-stage object detection models, such as R-FCN [21] and R-CNN [22], which sequentially perform target localization and recognition. These networks are highly accurate, albeit with the disadvantage of slower processing times. Such models are typically employed in scenarios where precision is paramount, and the speed of detection is a secondary concern. The second category includes single-stage methods, represented by the YOLO family of networks [23−26] and the SSD [27], which integrate object detection and localization into one step. This integration offers a balanced trade-off between detection speed and quality, making it suitable for applications where both factors are crucial. Recent advancements in this domain have seen significant enhancements, such as the YOLOv5 and the latest YOLOv7 model. When combined with UAV technology, these models provide efficient and precise detection capabilities, particularly in the tracking of endangered species, thereby advancing the field of automatic wildlife detection [28,29].

Recent advancements in wildlife monitoring have increasingly leveraged deep learning technologies. Zhao et al. [30] developed an enhanced YOLO network tailored for real-time wildlife detection in the Tongbiguan Nature Reserve, Yunnan, located in the tropical region of southwestern China. In their approach, they substituted the original YOLO backbone with a lightweight MobileNet architecture, facilitating rapid detection on CPU platforms. Wenhan et al. [31] refined this approach by introducing an enhancement algorithm based on YOLOv5s, which includes dataset enrichment, optimization of the channel attention mechanism, integration of the Swin Transformer, and implementation of the α-DOOU loss function. This modification has improved the accuracy and regression rate of wildlife detection, although it increased computational complexity and reduced inference speed. Further, Sun et al. [32] incorporated deformable convolutional network v2 (DCNv2) and WIoU into YOLOv7, enhancing feature extraction and learning capabilities. This adaptation addressed issues of low detection accuracy due to occlusion and blur in field environments but added to the computational and parameter complexity. Li et al. [33] focused on enhancing feature extraction and suppressing background interference in YOLOv7 by integrating O-ELAN and O-SPPCSPC modules with the CBAM. This significantly improved detection accuracy by reducing environmental interferences, though it necessitated a trade-off between real-time performance and accuracy, without exploring a more optimal balance between the two. While these improvements have enhanced the accuracy of wildlife detection, most have not been validated for use on UAV platforms, which often have limited computing resources. Additionally, these enhancements generally increase model complexity and parameter count, resulting in slower detection speeds and challenges in maintaining stable, long-term real-time monitoring.

## 2. Materials and methods

### 2.1. Study area and objects

**Fig 1:**
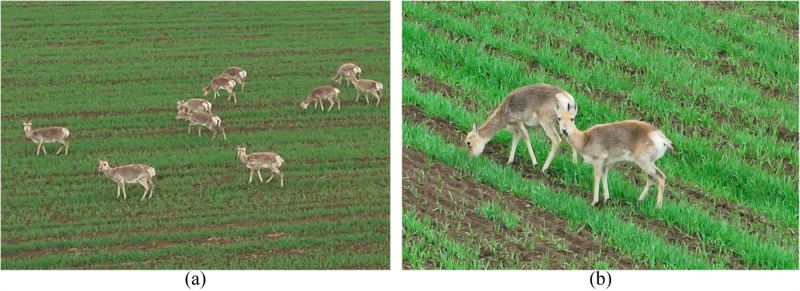
(a and b) High resolution image of Procapra przewalskii.

The dataset for this study was gathered in Haergai Town, located within Gangcha County of the Haibei Tibetan Autonomous Prefecture. Situated at an average elevation of 3,300 meters, this area exemplifies the plateau climate conditions typical of the region. Field research revealed that the Procapra przewalskii flourishes on the sandy grasslands adjacent to lake shores, identifying this region as a critical habitat for this globally endangered species. The significance of this locale extends beyond its unique geographical features, as it plays a crucial role in the conservation of the Procapra przewalskii and in ecological restoration efforts across the erosion-sensitive zones of the Qinghai Tibet Plateau. For data collection, an UAV was employed, operating at altitudes ranging from 20 to 50 meters. Over a span of three days, the research team successfully collected a substantial volume of video data, as depicted in Fig 1a and 1b. This visual data was instrumental in establishing two targeted databases, which have become vital resources in the ongoing efforts to effectively detect and monitor the populations of this species.

### 2.2. System overview

**Fig 2:**
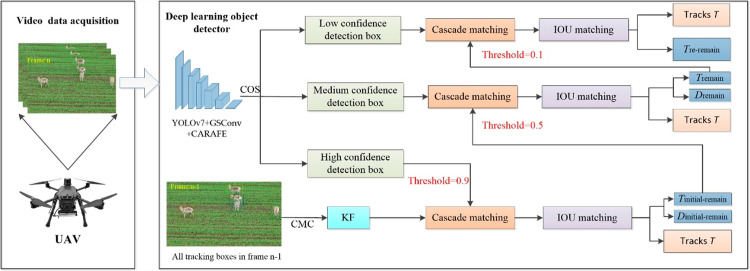
Overall framework of this study.

Fig 2 illustrates the MOT system designed for wildlife observation, which incorporates UAV equipped with high-definition cameras and advanced deep learning algorithms. This integration aims to enhance monitoring effectiveness in complex environments densely populated by Procapra przewalskii, thereby enabling more accurate status monitoring and improved protection of this species. Compared to traditional wildlife tracking methods, this system offers more efficient data collection and analysis, along with superior real-time performance. In this setup, footage from the UAV cameras serves as the primary data input. Initially, the YOLOv7 algorithm facilitates efficient object detection within the UAV monitoring system, ensuring rapid and precise identification and localization of Procapra przewalskii. This algorithm excels in determining spatial coordinates of targets within video sequences and maintaining high accuracy in real-time implementation. Following detection, the Deep SORT algorithm is employed to monitor the precise location of the identified targets, ensuring continuous tracking of the animals detected by YOLOv7, even in complex environments. This enhancement significantly improves the system’s ability to track the movements of Procapra przewalskii effectively. The application of this visual tracking technology is crucial not only for conservation support but also for maintaining ecosystem equilibrium, facilitated by accurate and comprehensive information on the behaviors and movements of Procapra przewalskii.

Within the TBD framework, the effectiveness of tracking systems critically depends on the detection algorithms utilized. To improve the precision of these detections, the YOLOv7 model in our system has been enhanced with the CARAFE method, replacing the traditional nearest neighbor interpolation. This adaptation allows for the capture of more comprehensive and detailed target information, leading to improved detection outcomes and more precise localization of each target. Additionally, to accommodate the limited computational resources typical of UAV, GSConv convolutions have been integrated into the YOLOv7 neck network. This modification significantly reduces the computational burden, facilitating more efficient processing, which is particularly beneficial for UAV monitoring Procapra przewalskii in complex and demanding environments. Furthermore, enhancements to the tracking algorithm include the development of a CMC module. This module is designed to refine KF predictions, particularly correcting for inaccuracies caused by rapid movements of UAV cameras in high-altitude settings. It effectively reduces tracking failures associated with horizontal, vertical, and rotational movements, enhancing the reliability of monitoring Procapra przewalskii. To address occlusions, a COS has been established. This strategy adjusts the matching thresholds based on varying confidence levels of detection frames to better handle occlusions, thereby enhancing detection accuracy and the overall efficiency of the MOT system. These strategic enhancements are pivotal for consistent and reliable wildlife tracking, ensuring optimal system performance in practical field applications. Equipped with this advanced MOT system, UAV can conduct real-time and extensive area monitoring in challenging environments, optimizing resource use and minimizing disturbance to wildlife.

### 2.3. Detector

YOLOv7 [34] represents an advanced iteration within the YOLO series, renowned for its real-time object detection capabilities. This series characteristically approaches object detection as a single regression problem, aiming to predict both the position (bounding box) and category of the object in one forward pass. Building on the advancements of YOLOv4, YOLOv7 has been further enhanced to improve both accuracy and inference speed, making it particularly adept for real-time object detection applications.

**Fig 3:**
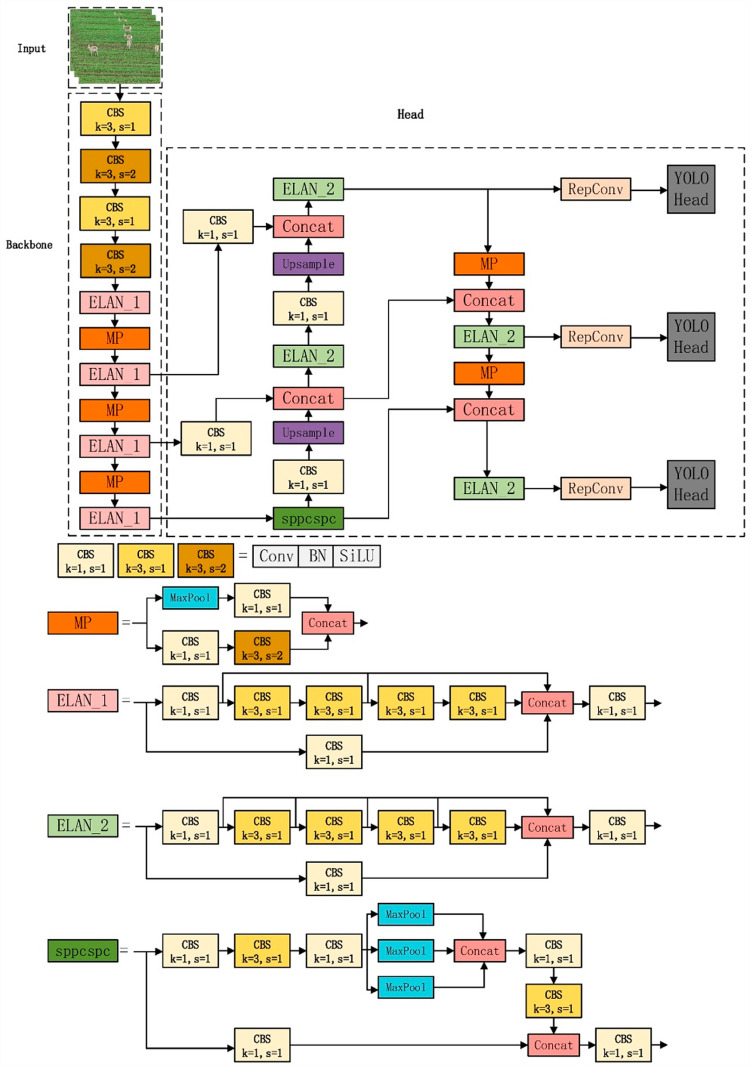
Network structure of the YOLOv7 model.

In 2022, the YOLOv7 model was innovatively developed by Chien-Yao Wang and Alexey Bochkovskiy. It incorporates the Extended High-Efficiency Layer Aggregation Network (E-ELAN) [35] and utilizes advanced techniques such as model scaling and re-parameterization strategies derived from cascading models [36,37]. This development strategically balances the trade-offs between detection efficiency and accuracy, marking a critical advancement in the field. As illustrated in Fig 3, the YOLOv7 architecture is methodically structured into four primary components: the Input module, the Backbone network, the Head network, and the Prediction Network Head. Each component is meticulously designed to process input data through progressive stages of the network, significantly enhancing the model’s overall detection performance and speed.

In this investigation, we have implemented modifications to the YOLOv7 architecture by incorporating GSConv convolutions in the neck network. This integration effectively preserves a higher level of detail while significantly reducing the computational load. Such a strategic inclusion is crucial for retaining critical data that is often lost in traditional convolution processes. Additionally, we have replaced conventional nearest neighbor interpolation with the CARAFE module during the up-sampling process. This modification not only minimizes the loss of feature data but also enhances the efficiency and precision of the up-sampling mechanism in YOLOv7.

#### 2.3.1. GSConv convolution.

**Fig 4:**
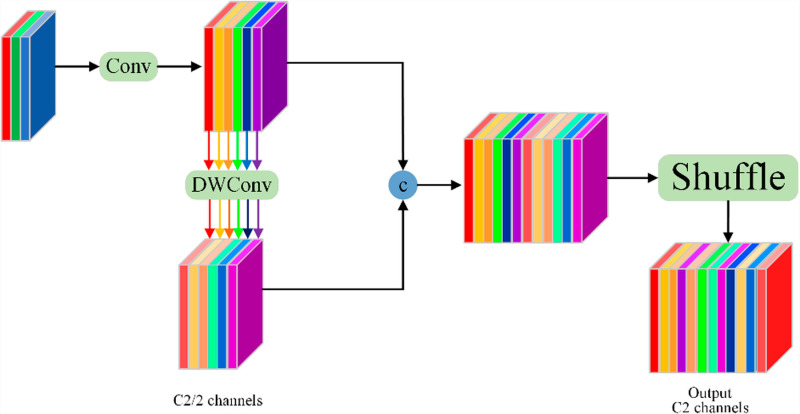
GSConv module.

Although the implementation of depthwise separable convolution [38] effectively reduces both the computational load and parameter count within the model, it processes the information independently across each channel during the convolution phase. This approach, unfortunately, can lead to suboptimal feature interaction and integration across channels. To address the limitations of isolated channel information inherent in depthwise separable convolution, Li et al. [39] have introduced GSConv convolution, as depicted in Fig 4. This technique initially reduces the number of input channels, C1, to half of the output channels, C2, using standard convolution methods. It then employs depthwise separable convolution to process the modified channel quantity. Finally, it combines the outputs from the standard and depthwise separable convolutions through a Shuffle operation, ensuring a more cohesive fusion of information across channels.

As deep neural networks process feature fusion, semantic information propagates in a downward trajectory, undergoing alterations across the dimensions of feature maps—specifically, height, width, and channel count. These dynamic changes can inadvertently result in the diminution of semantic information, potentially undermining the predictive accuracy of the model. To mitigate these issues, our research integrates GSConv convolution into both the Neck layer and the ELAN module of the network architecture. This methodological enhancement effectively reduces the network’s parameter count while simultaneously preserving the accuracy of detection.

#### 2.3.2. CARAFE to improve feature fusion performance.

In the realm of network models used for image classification and target detection, the integration of up-sampling techniques within the architecture of feature pyramids is essential. These up-sampling strategies are generally categorized into two main types. The first type includes linear interpolation methods such as nearest neighbor interpolation and bilinear interpolation. Although these methods are effective for enhancing local features, they tend to neglect the comprehensive semantic information present across the entire feature map, focusing instead only on immediate neighboring data points.

Furthermore, the linear interpolation techniques currently employed exhibit a limited sensory scope, which inadequately captures the global characteristics of images. An alternative, inverse convolutional up-sampling, expands image dimensionality through convolution processes. However, this method uniformly applies the same convolutional kernel across the entire image, thus limiting its ability to effectively discern local variations. Additionally, it leads to an increased parameter count within the model. To address these shortcomings of conventional up-sampling methods, this paper introduces the CARAFE [40] up-sampling operator in YOLOv7. Characterized by its minimal redundancy and high-speed operation, the CARAFE operator represents a significant improvement over nearest-neighbor interpolation. It is distinguished by its ability to aggregate semantic information across a broader receptive field and utilizes an adaptively generated kernel to efficiently up-sample distinct feature points. This innovative approach greatly reduces the loss of feature information typically associated with up-sampling processes and preserves the integrity of feature data throughout the model.

**Fig 5:**
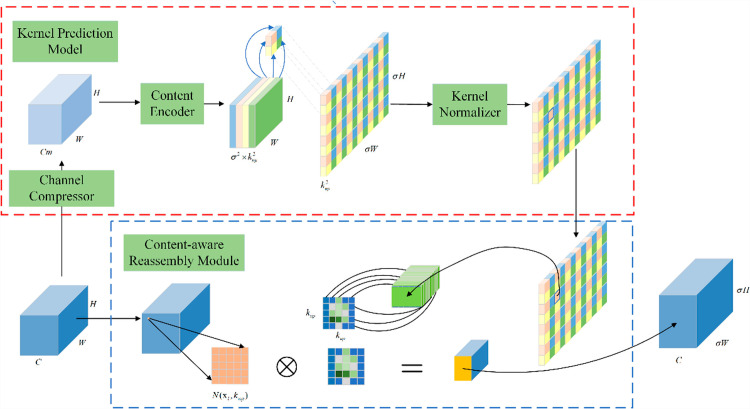
CARAFE structure.

The structure of the up-sampling operator CARAFE is depicted in Fig 5. The CARAFE up-sampling method comprises two main components: up-sampling kernel prediction and content-aware recombination. Initially, the up-sampling kernel is predicted based on the content at each target location. Subsequently, the feature map channel, with size compressed to $C_{m}$ , is processed. The up-sampling factor used is *σ*, which is set to 2 in this study.

The up-sampling kernel size is $k_{up}\times k_{up}$. This process transforms the channel into $\sigma^{2}\times k_{up}^{2}$ through content encoding, then expands the channel in the spatial dimension, and finally normalizes the predicted up-sampling kernel by applying the softmax function to ensure the convolution kernel has a total weight of 1. The working principle of the content-aware reconstruction part maps each position in the output feature map back to the input feature map; this process is achieved through a dot product operation between the predicted up-sampling kernel and the corresponding original feature map region. Consequently, the process outputs a feature map of size $\sigma_{H}\times \sigma_{W}\times C$. By predicting the up-sampling kernel and completing feature recombination based on the kernel, the perceptual information of the input feature map is fully utilized to achieve efficient up-sampling. In this study, by employing the CARAFE up-sampling method instead of the traditional nearest neighbor interpolation in the original network, the detailed features of the target were significantly restored, thereby greatly enhancing detection accuracy.

### 2.4. Tracker

#### 2.4.1. Objects tracking method.

Deep SORT is an algorithm utilized for MOT, building on the foundational SORT algorithm. SORT itself is an efficient online multi-target tracking method that employs a KF to estimate the state of targets and the Hungarian algorithm to resolve data association challenges. Building on this, Deep SORT incorporates deep learning models to enhance the accuracy of object association, particularly in scenarios where the appearance of objects is similar or obscured. The Deep SORT algorithm uses the KF to predict the trajectories of moving objects and combines this with appearance features extracted from a deep Convolutional Neural Network (CNN) to improve target tracking capabilities. The subsequent section provides a detailed discussion of these components:

An eight-dimensional state space defined by $\left(x,y,\gamma ,h,\dot{x},\dot{y},\dot{\gamma },\dot{h}\right)$ is employed to describe the target’s state at a specific time. Here, (x, y) denotes the coordinates of the box center, *γ* represents the aspect ratio of the detection bounding box, h denotes the height of the detection bounding box, and $（\dot{x},\dot{y},\dot{\gamma },\dot{h}）$ represents the relative velocity in the image space （x,y,γ,h）. Additionally, the KF of the uniform model, alongside the linear observation model, is utilized to update the target state.

In this algorithm, the integration of motor information with the visual appearance features of targets is crucial for accurately correlating and matching detected objects. To assess motion dynamics, the algorithm utilizes the Mahalanobis distance to evaluate the similarity between the detected bounding boxes produced by the detection systems and those predicted by the KF. This measurement is essential for determining the degree of correlation between the current and predicted states of a target. The computational approach for calculating the Mahalanobis distance is specified as follows:


$$d^{\left(1\right)}\left(i,j\right)={\left(d_{j}-y_{i}\right)}^{T}s_{i}^{-1}\left(d_{j}-y_{i}\right)$$ (1)

where $d^{\left(1\right)}\left(i,j\right)$ represents the measurement on motion state between the i-th predicted bounding box and the j-th detection bounding box, $d_{j}$ represents the j-th prediction bounding box, $y_{i}$ represents the i-th detection bounding box, and $s_{i}$ represents the covariance matrix between the i-th and the mean of the predicted bounding box.

In the application of the Deep SORT algorithm, specialized networks dedicated to feature extraction are employed to gather relevant information from both detected and predicted bounding boxes, focusing on the appearance characteristics of targets. This process is critical in enhancing the tracking accuracy of the algorithm. Furthermore, the correlation between detected and projected bounding boxes is quantified using cosine distance, derived from the outputs of these feature extraction networks. This cosine distance effectively measures the similarity between the appearance features of the bounding boxes. The specific methodology for calculating this cosine distance is outlined as follows:


$$d^{\left(2\right)}\left(i,j\right)=\min\left\{1-r_{j}^{T}r_{k}^{\left(i\right)}|r_{k}^{\left(i\right)}\in R_{i}\right\}$$ (2)

where $r_{j}$ represents the appearance feature descriptor of the detection bounding box $d_{j}$, and the restriction condition is $\left|r_{j}\right|=1$. $d^{\left(2\right)}\left(i,j\right)$ represents minimum cosine distance from the i-th detected bounding box to the j-th predicted bounding box. When $d^{\left(2\right)}\left(i,j\right)$ is below the threshold value, it is considered that the two bounding boxes match with each other correctly. For every matched track, a graph gallery $R_{i}={\left\{r_{k}^{\left(i\right)}\right\}}_{k=1}^{L_{k}}$ is created. It can store the last n descriptor (n is a setable parameter).

The Mahalanobis distance is highly effective for precise short-term target localization and significantly enhances both prediction and matching accuracy. However, in scenarios where the target is obscured or missing for extended periods, the cosine distance becomes crucial in re-establishing the target’s identity. To capitalize on the strengths of both metrics, this study integrates the Mahalanobis distance with the cosine distance through a linear weighting scheme. This hybrid approach serves as the definitive metric for evaluating target correspondence in the implemented system. The formulation for this combined measurement is detailed in the following section:


$$c_{i,j}=\lambda d^{\left(1\right)}\left(i,j\right)+\left(1-\lambda \right)d^{\left(2\right)}\left(i,j\right)$$ (3)

where *λ* is a setting parameter, $c_{i,j}$ is the final measurement value.

In scenarios where a target remains obscured for prolonged periods without positional updates, the KF, operating with an enlarged covariance matrix, tends to produce less accurate predictions. This condition often complicates the tracking process, as the predicted path may deviate from the target’s actual trajectory. To effectively address this challenge, this study introduces a method where trajectories that disappear are classified as ‘missing’. These trajectories are then meticulously monitored, and upon their subsequent detection, they are reclassified and reintegrated into the system as ‘matching’ trajectories, thereby reestablishing continuity with the tracked target.

#### 2.4.2. Camera motion compensation.

Successfully tracking the Procapra przewalskii in diverse and complex environments poses a substantial challenge. The KF algorithm, which utilizes linear system state equations for enhanced precision, is predominantly used for optimal estimation of system states in tracking endeavors. This algorithm predicts the location of the target and integrates these predictions with real-time observed location data to formulate the most precise estimate of the target’s position. Currently, a wide array of sophisticated multi-target tracking strategies employ an 8-tuple configuration of KF state vectors, meticulously designed to capture various dynamics of moving objects. The configuration is outlined as follows:


$$D=\left[x,y,\gamma ,h,v_{x},v_{y},v_{\gamma },v_{h}\right]$$ (4)

In the Deep SORT tracking framework, the state vector D is meticulously composed of several key parameters: u and v represent the horizontal and vertical centroid coordinates of the prediction frame, respectively, while *γ* and hhh detail the width and height of that frame. Additionally, the derivatives $v_{x}$, $v_{y}$, $v_{\gamma }$, and $v_{h}$ quantify the rates of change in each corresponding dimension—x, y, *γ*, and h. To enhance tracking accuracy over time, Deep SORT utilizes Q and R as functions to systematize the estimation of key elements, aligning them with predefined metrics. Continuous recalibrations of the KF ‘s state vector are essential to maintain precision, particularly focusing on the covariances of process noise (Qn) and measurement noise (Rn). These covariances are rigorously defined in Equations (5) and (6):


$$\begin{array}{l} Q_{n}=diag({\left(\sigma_{p}{\hat{\gamma }}_{n-1|n-1}\right)}^{2},{\left(\sigma_{p}{\hat{h}}_{n-1|n-1}\right)}^{2},\\ {\left(\sigma_{p}{\hat{\gamma }}_{n-1|n-1}\right)}^{2},{\left(\sigma_{p}{\hat{h}}_{n-1|n-1}\right)}^{2},\\ {\left(\sigma_{v}{\hat{\gamma }}_{n-1|n-1}\right)}^{2},{\left(\sigma_{v}{\hat{h}}_{n-1|n-1}\right)}^{2},\\ {\left(\sigma_{v}{\hat{\gamma }}_{n-1|n-1}\right)}^{2},{\left(\sigma_{v}{\hat{h}}_{n-1|n-1}\right)}^{2}) \end{array}$$ (5)

$$\begin{array}{l} R_{n}=diag({\left(\sigma_{m}{\hat{\gamma }}_{n|n-1}\right)}^{2},{\left(\sigma_{m}{\hat{h}}_{n|n-1}\right)}^{2},\\ {\left(\sigma_{m}{\hat{\gamma }}_{n|n-1}\right)}^{2},{\left(\sigma_{m}{\hat{h}}_{n|n-1}\right)}^{2}) \end{array}$$ (6)

Throughout the tracking sequence, the rapid movements of Procapra przewalskii present significant challenges for the UAV camera, which must execute intricate aerial maneuvers to effectively track the swiftly moving target. This activity often leads to misalignment of the target across consecutive frames, complicating the tracking process. Additionally, the intrinsic movement patterns of the Procapra przewalskii, which deviate from the linear motion assumptions fundamental to the KF ‘s functionality, add further complexity. As a result, these deviations undermine the accuracy of the position frames predicted by the KF, frequently leading to erroneous tracking outcomes.

**Fig 6:**
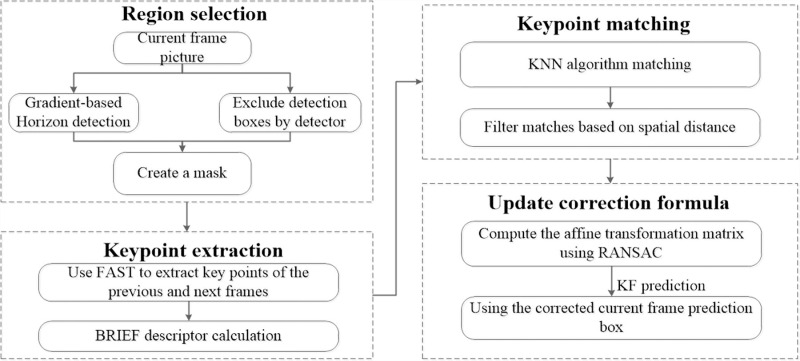
The execution process of CMC module.


To significantly enhance the prediction accuracy of the KF and mitigate errors arising from UAV camera movements, this paper introduces a newly developed CMC module. As illustrated in Fig 6, the module comprises four primary steps. The initial step involves identifying static regions within the image that are suitable for anchoring key points, ensuring a stable basis for subsequent analyses. Subsequently, the module employs an advanced level detection strategy that leverages image gradients to effectively prevent mismatches, particularly those caused by surface reflections. The approach to horizon detection is articulated through a mathematical formulation, specified as follows:

$$H={\text{argmax}}_{y}(\frac{1}{w}\overset{w}{\underset{x=1}{\sum }}\left|G_{y}\left(x,y\right)\right|)$$ (7)

In the formulation presented in Equation (7), the symbol H is used to denote the vertical location of the horizontal line within the image, determined specifically as the row exhibiting the highest average absolute gradient value. The function $G_{y}\left(x,y\right)$ quantifies the gradient in the vertical direction at the point $\left(x,y\right)$ within the image. Furthermore, W, representing the width of the image, serves as a normalizing factor in the computation process. The formula $\overset{w}{\underset{x=1}{\sum }}|G_{y}\left(x,y\right)|$ is employed to accumulate the absolute values of the gradients along each row. This accumulated sum is subsequently averaged over the width W of the image, which effectively standardizes the measurement of gradient intensity per row, providing a clearer metric for identifying significant horizontal lines based on gradient variations.

Regions identified by the YOLOv7 detector that contain moving targets are systematically excluded, effectively isolating static background areas for key-point detection. This methodology ensures that only static elements are included in further analysis, thus enhancing the definition and focus of background features.

Subsequently, key points within the static background are identified and correlated across successive frames from the designated mask region using the FAST corner detection algorithm, which encodes these points with the compact BRIEF descriptor, a component of the ORB approach. This ensures processing efficiency while maintaining real-time capabilities. During the matching phase, the KNN algorithm is employed, selectively filtering matches by assessing their spatial distances. This method effectively discards matches that are spatially distant, thereby ensuring high-quality matching and precise capture of essential background details.

Subsequently, the Randomized Sampling Consistency Algorithm (RANSAC) computes the affine transformation matrix representing the background motion. This matrix, denoted as $A_{n|n-1}$, represents the coordinate transformation from the previous frame to the current frame and can be expressed as follows:


$$A_{n|n-1}=[M_{2\times 2}|T_{2\times 1}]$$ (8)

where $M_{2\times 2}$ denotes the transformation matrix for the rotation and scaling of the coordinate system, and $T_{2\times 1}$ denotes the transformation matrix for the translation part of the coordinate system.

In order to align the dimensions of the state vectors, an extended version of the matrix ${\hat{M}}_{n|n-1}$ is configured as an 8 ×  8 matrix. This extension is achieved by diagonalizing the matrix, which can be represented as follows:


$${\hat{M}}_{n|n-1}=diag\left\{M_{2\times 2},M_{2\times 2},M_{2\times 2},M_{2\times 2}\right\}$$ (9)

The extended version ${\hat{T}}_{n|n-1}$ is configured as an 8 × 1 vector as shown in the following equation:


$${\hat{T}}_{n|n-1}=\left[\begin{array}{c} T_{2\times 1}\\ 0\\ 0\\ 0\\ 0\\ 0\\ 0 \end{array}\right]$$ (10)

Using the transformation matrix derived from Eqs. (9) and (10), the predicted state vector is updated as shown in the following equation:


$${\hat{x}}_{n|n-1}^{\text{'}}={\hat{M}}_{n|n-1}{\hat{x}}_{n|n-1}+{\hat{T}}_{n|n-1}$$ (11)

where ${\hat{x}}_{n|n-1}$ denotes the KF prediction state before CMC, and ${\hat{x}}_{n|n-1}^{\text{'}}$ denotes the KF prediction state after updating the correction.

In addition, the covariance matrix is updated according to the changes in the state vector, which can be expressed as:


$$P_{n|n-1}^{\text{'}}={\hat{M}}_{n|n-1}P_{n|n-1}{\hat{M}}_{n|n-1}^{T}+Q_{n}$$ (12)

where $P_{n|n-1}$ denotes the covariance matrix of the KF prediction before CMC and $P_{n|n-1}^{\text{'}}$ denotes the covariance matrix after compensation.

For the state estimation update at time t, the Kalman gain $K_{n}$ is initially computed based on the prediction error covariance $P_{n|n-1}^{\text{'}}$, the observation matrix $H_{n}$, and the observation covariance noise $R_{n}$. Then, the model’s prediction ${\hat{X}}_{n|n-1}^{\text{'}}$ is combined with the residual observation term $z_{n}-H_{n}{\hat{x}}_{n|n-1}^{\text{'}}$ to obtain the corrected state estimate ${\hat{X}}_{n|n}$. Finally, the error covariance $P_{n|n}$ is adjusted according to the Kalman gain as shown in the following equation:


$$K_{n}=P_{n|n-1}^{\text{'}}H_{n}^{T}{\left(H_{n}P_{n|n-1}^{\text{'}}H_{n}^{T}+R_{n}\right)}^{-1}$$ (13)

$${\hat{x}}_{n|n}={\hat{x}}_{n|n-1}^{\text{'}}+K_{n}\left(z_{n}-H_{n}{\hat{x}}_{n|n-1}^{\text{'}}\right)$$ (14)

$$P_{n|n}=\left(1-K_{n}H_{n}\right)P_{n|n-1}^{\text{'}}$$ (15)

To effectively address the dynamic challenges posed by UAV camera movements in wildlife monitoring, the CMC module has been specifically engineered to enhance the KF. This improvement facilitates more precise estimations of Procapra przewalskii movements across consecutive video frames. The refined method tracks not only the inherent movement patterns of Procapra przewalskii but also compensates for the instabilities caused by UAV operational dynamics. As a result, it ensures improved and reliable tracking of the target within the video sequences, substantially enhancing the robustness and accuracy of the wildlife tracking system.

#### 2.4.3. Confidence optimization strategy.

To address challenges related to target occlusion and fluctuating detection confidence levels, a novel COS was developed, diverging significantly from methodologies previously documented in references [39,40]. This innovative strategy retains almost all detection boxes, stratifying them into high, medium, and low confidence categories based on varying detection score thresholds. This classification system enables the application of specific matching thresholds for boxes at each confidence level, facilitating a more refined approach to cascaded matching with predicted trajectories. High confidence detection boxes, typically reliable, require the application of a high matching threshold to meet stringent matching criteria. Conversely, medium confidence boxes, affected by partial occlusions, necessitate a slightly lower matching threshold. This adjustment allows for minor positional shifts, enhancing the algorithm’s stability in dynamic environments. For detection boxes categorized as low confidence, often resulting from occlusion or poor lighting, the matching threshold is significantly reduced. This reduction is crucial for mitigating the loss of target information, essential for maintaining continuous tracking in complex environments. The system thus provides an opportunity to correct errors by increasing the selection of potential matches. To this end, adaptive and iterative adjustments and enhancements are made to the trajectory associations based on prior matchings. This incremental enhancement of confidence across all levels aims to improve the overall match quality, thereby stabilizing and enhancing the reliability of target tracking in cluttered environments.

In addition to addressing low-confidence detection boxes that experience significant feature information loss, the COS effectively mitigates the issue of false positives associated with these boxes. Under the COS framework, such boxes are assigned the lowest matching priority and are only considered if higher-confidence boxes, both high and medium, fail to align with any tracks. This prioritization minimizes the impact of false positives on the overall tracking performance, significantly reducing their interference. Furthermore, the strategy enhances the system’s ability to maintain continuous and effective tracking by avoiding missed matches caused by occlusion challenges. This methodology is particularly advantageous in complex tracking environments, where it dynamically adjusts the matching criteria to adapt to rapidly changing and challenging scenarios.

## 3. Experiments and results

For the experimental setup, an NVIDIA GeForce RTX 4070 graphics card was employed, supported by the CUDA 11.1 and CUDNN-V8.0.4.30 GPU acceleration libraries. The computational environment was configured with PyTorch version 1.8.1 and Python 3.8 to develop a detection model that combines high stability with rapid convergence. Hardware specifications are detailed in Table 2. To optimize the training process, the Adam optimizer was chosen for its efficiency in handling large datasets, with parameters set to 120 iterations and a weight decay of 0.0005. The batch size was set to 32 to balance computational load and memory utilization. The model training parameter configurations are provided in Table 3.

**Table 2:** Experimental environment.

Name	Configuration information
Operating system	Windows 10
Graphics card	NVIDIA GeForce RTK 4070
CPU	AMD Ryzen 9 5900X
GPU Acceleration Library	CUDA 11.1 CUDNN-V8.0.4.30
Software	Python 3.8, Pycharm 2020.1

**Table 3:** Model training parameter configuration.

Name	Configuration information
Batch size	32
Initial learning rate	0.03
Final learning rate	0.0001
optimizer	Adam
weight decay	0.0005
Epochs	200

### 3.1. Dataset

In the designated research area, a dataset comprising 20 video sequences of Procapra przewalskii was selected for detection and tracking studies. Each dataset includes 10 unique video clips, providing comprehensive coverage of the species’ behavior and movement. These video sequences serve as benchmarks for accurately identifying locations and tracking the movements of Procapra przewalskii, analyzed rigorously using the YOLOv7 model.

To enhance the detection and identification of Procapra przewalskii, with a particular emphasis on resolving fur texture details, a specialized dataset was created. This dataset, consisting of 3,000 images, was extracted from 10 selected video sequences for object detection. The images were systematically divided into training, validation, and testing sets with a distribution ratio of 70%, 20%, and 10%, respectively. The YOLOv7 model was utilized to refine parameters, aiming to improve the accuracy and stability of the detection process.

To optimize the performance of the Deep SORT algorithm for effective tracking and monitoring of wildlife, particularly Procapra przewalskii, a detailed dataset was constructed. Frame images were captured from 10 video sequences. The DarkLabel software is utilized for annotating these images, storing them in XML format to form the core of the target tracking dataset. This structured approach effectively organizes the data and prepares it for comprehensive analytical use in tracking applications. The dataset was then strategically divided, allocating 70% for training to develop robust tracking models, 20% for testing to evaluate performance under varied conditions, and 10% for validation to fine-tune and confirm the accuracy and reliability of the tracking system across diverse scenarios.

### 3.2. Evaluation indicators

This study employed the following evaluation criteria to assess the performance of the algorithm.

In target detection, the model typically detects multiple classes of targets, with each class generating a precision-recall (PR) curve, from which an average precision (AP) value is derived. AP represents a scalar measure of the area under the PR curve. The mean average precision (mAP) is calculated as the average of the AP values across all classes, as defined by Eq. (16):


$$mAP=\frac{1}{class\_number}\sum_{1}^{class\_number}AP$$ (16)

MOT accuracy (MOTA) serves as an intuitive gauge for evaluating the performance of object detection and trajectory maintenance, which could be computed with the following equation:


$$MOTA=1-\frac{\sum_{t}FN+FP+IDSW}{\sum_{t}GT_{t}}$$ (17)

MOT precision (MOTP) was adopted to judge the positioning precision.


$$MOTP=\frac{\sum_{t,i}d_{t,i}}{\sum_{t}c_{t}}$$ (18)

The IDF1 metric, defined by Eq. (19), is the Identification F1 score which evaluates the accuracy of identity matches. This score comprises IDTP, the count of accurate identity matches, while IDFP and IDFN denote the counts of erroneous matches and failures to match identities, respectively.


$$IDF1=\frac{2\times IDTP}{2\times IDTP+IDFP+IDFN}$$ (19)

FP represents the count of error alerts related to the trajectory of incorrectly predicted objects.

FN reflects the count of missed detections and undetected tracking objects.

IDs denotes the count of instance in which assigned IDs change during tracking.

### 3.3. Evaluation of benchmarks

In this study, the YOLOv7 model was enhanced by incorporating the CARAFE module to replace the original nearest neighbor interpolation method, enabling the capture of more detailed and comprehensive target information. Additionally, GSConv convolution was introduced in place of traditional convolution within the YOLOv7 neck network, significantly reducing the algorithm’s computational burden. To evaluate the efficacy of these enhancements, a series of comparative tests were conducted between the improved YOLOv7 model and other YOLO series models, using the Procapra przewalskii dataset specifically created for this purpose. Consistent experimental conditions were maintained, with all input image dimensions set to 640 ×  640. As shown in Table 4, the results demonstrate that the improved YOLOv7 model significantly outperforms its predecessors and contemporaries, including YOLOv3, YOLOv5s, and YOLOv8, in detection performance. The enhanced model achieves the highest mAP of 93.8% while maintaining the lowest parameter count of 11.7M. These findings validate the effectiveness of the proposed modifications and highlight the robustness and reliability of the improved algorithm for UAV-based wildlife detection applications.

**Table 4:** Comparison with different detection methods.

Model	mAP ↑ (%)	Precision ↑ (%)	Recall ↑ (%)	F1 ↑ (%)	Parameters ↓ (M)
YOLOv3	63.5	63.2	65.7	65.2	17.8
YOLOv5s	87.3	88.1	89.5	87.6	14.5
YOLOv8	91.4	91.3	93.5	93.8	36.5
YOLOv7	89.1	89.8	90.1	90.3	13.5
Ours	93.8	92.2	95.0	94.5	11.7

**Fig 7:**
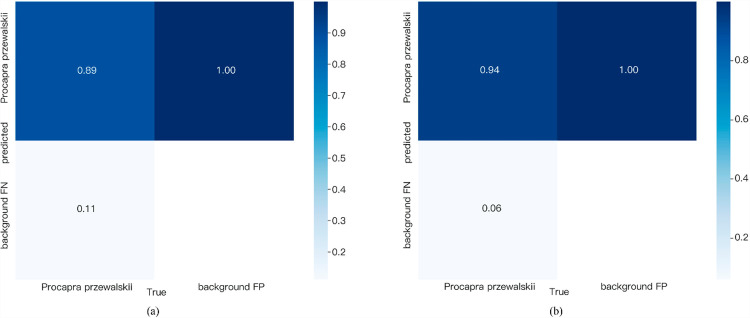
Confusion matrix. **(a)**YOLOv7; **(b)** Ours.

In Fig 7, we evaluated the performance of our improved YOLOv7 model and the original YOLOv7 model, both of which were trained on the same dataset. The confusion matrix of the two models is shown in Fig 7. It can be seen that our improved YOLOv7 model performs better in identifying Procapra przewalskii, with 94% of Procapra przewalskii predicted correctly, while YOLOv7’s prediction accuracy is 89%. This highlights the effectiveness of our improved YOLOv7 model in terms of recognition accuracy.

**Fig 8:**
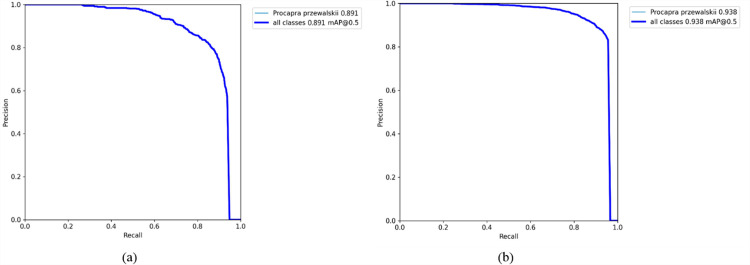
Comparison of P-R curves. **(a)** YOLOv7 model; **(b)** Ours.

Fig 8 shows the comparison of the P-R curves between the original YOLOv7 model and our improved YOLOv7 model, indicating that our improved YOLOv7 model performs better.

**Fig 9:**
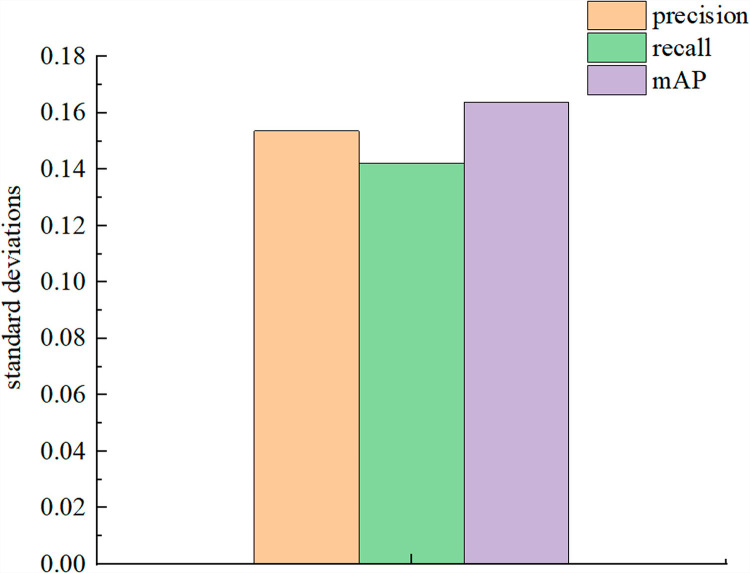
The standard deviation of the training results for each round of our improved YOLOv7 model.

Fig 9 shows the precision, recall, and standard deviation of each round of training results for our model, which are 0.1536, 0.1422, and 0.1639, respectively. These results demonstrate the stability of the model.

**Fig 10:**
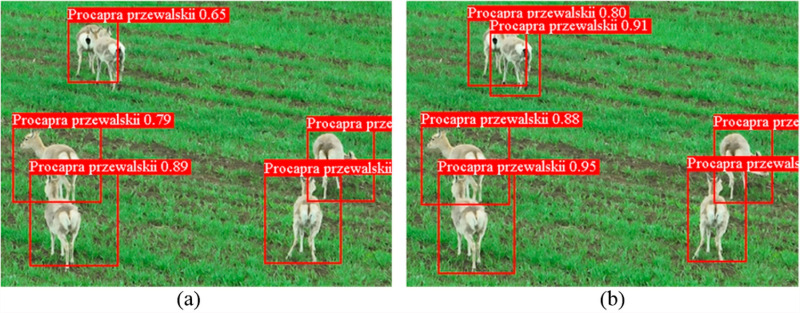
Comparison of some test results. **(a)** Original YOLOv7. **(b)** Improved YOLOv7.

Fig 10 delineates a comparative analysis of detection efficacy between the conventional YOLOv7 model and its enhanced version. It is evident from the analysis that the modified YOLOv7 demonstrates superior detection capabilities and elevated accuracy. This enhancement conclusively substantiates the effectiveness of the improvements made to the model.

To assess the innovativeness and dependability of our newly developed UAV multi-target tracking system for wildlife observation, we conducted comparative analyses with established multi-target tracking methodologies. While other systems within the TBD paradigm typically employ the standard YOLOv7 model for detection, our approach integrates an enhanced version of the YOLOv7 model. This modification aims to demonstrate the superior effectiveness of our comprehensive system design. As indicated in Table 5, our evaluation included a range of prevalent tracking methods such as SORT, Deep SORT, ByteTrack, BoT-SORT, JDE, and FairMOT, showcasing our method’s comparative advantages.

**Table 5:** Comparison of tracking results between our method and other mainstream methods.

Tracker	MOTA ↑ (%)	MOTP ↑ (%)	IDF1 ↑ (%)	FP ↓	FN ↓	IDs ↓	FPS ↑
SORT	50.6	66.8	49.5	12867	9632	653	38.6
Deep SORT	62.3	75.2	60.0	10358	6053	228	16.9
ByteTrack	61.6	76.7	60.3	12366	4255	236	31.2
BOT-SORT	63.5	78.2	63.8	11658	3532	208	13.5
JDE	62.8	76.9	61.2	10358	5865	396	25.8
FairMOT	63.8	78.3	63.9	12163	3275	269	27.3
Ours	69.3	78.9	65.8	10162	2615	162	25.6

SORT demonstrates robust real-time tracking capabilities; however, it often lacks sufficient accuracy, leading to the loss of targets. Deep SORT enhances this aspect by evaluating the similarity of appearance features, yet it still encounters difficulties with small or partially obscured targets in datasets. These challenges can obscure object distinctions, thus reducing the method’s tracking precision. ByteTrack attempts to improve accuracy by conducting secondary matching on targets with low confidence, while maintaining a high frame rate per second (FPS). However, its performance is compromised by environmental variables such as changes in lighting and physical deformations of targets, which impact its overall stability. BoT-SORT incorporates considerations for camera motion effects on tracking but lacks tailored optimizations for specific scenarios, which affects its real-time operational efficiency. Meanwhile, JDE extracts Re-ID information, represented as low-dimensional vectors, from both detection frames and the objects within them. The anchor points used by anchor-based detectors, however, may not be optimal for learning precise Re-ID information. This can lead to a single object being recognized at multiple points, thus introducing significant ambiguity into the network. Furthermore, the FairMOT algorithm integrates both detection and appearance feature extraction directly within its network architecture. This integration creates competition among the network components, which can increase the frequency of target identity switches during the tracking process. Notably, our approach integrates the GSConv, CARAFE, CMC, and COS modules, leading to a marked improvement in tracking accuracy on our custom Procapra przewalskii dataset, achieving an operational speed of 25.6 FPS. This integration showcases our method’s capability to deliver superior tracking performance under varied and challenging conditions.

### 3.4. Ablation experiment

The detection result format of YOLOv7 is inherently compatible with Deep SORT, reducing integration workload. Detection boxes generated by YOLOv7 can be directly utilized for tracking and associating inputs in Deep SORT, eliminating the need for significant adjustments to input and output configurations. The YOLOv7 + Deep SORT configuration offers flexibility for various scenarios, with performance optimization achievable by adjusting parameters or replacing specific modules, such as GSConv, CARAFE, CMC, and COS. Consequently, this study selected YOLOv7 + Deep SORT as the baseline model.

To validate the performance improvements achieved by the module introduced in this research, a comprehensive series of ablation studies was conducted using YOLOv7 and Deep SORT, as detailed in Table 6. These experiments were systematically designed to quantify the module’s contributions to enhancing the accuracy and robustness of wildlife tracking systems under diverse environmental conditions.

**Table 6:** Ablation study of different improved methods.

Baseline	GSConv	CARAFE	CMC	COS	MOTA ↑ (%)	IDF1 ↑ (%)	IDs ↓
√					62.3	60.0	228
√	√				63.1	60.8	223
√	√	√			63.8	61.3	219
√	√	√	√		66.2	63.4	195
√	√	√	√	√	69.3	65.8	162

As shown in Table 6, the integration of the GSConv and CARAFE modules has significantly enhanced the accuracy of MOT. The GSConv module improves convolution efficiency and generates feature maps with more effective feature representation. Following the introduction of GSConv, the MOTA and MOTP scores of the model increased by 0.8%, respectively. Additionally, the CARAFE module enhances the quality of up-sampling and improves detection accuracy by incorporating a content-aware feature recombination mechanism. With the inclusion of CARAFE, the MOTA and MOTP scores improved by 0.7% and 0.5%, respectively. These results highlight the critical role of advanced detectors within the framework of TBD-based tracking systems. Moreover, the introduction of the CMC module has significantly improved various tracking performance metrics. Specifically, the MOTA score increased by 2.4%, the IDF1 score improved by 2.1%, and the number of identity switches decreased by 24. These results indicate that the CMC module effectively enhances tracking performance by correcting errors in KF predictions caused by camera movement, particularly in scenarios with severe camera shake. Furthermore, the COS module addresses variations in confidence levels by applying different matching thresholds for detection frames with varying confidence levels. This approach increased the MOTA score to 69.3% and the IDF1 score to 65.8%, while further reducing the frequency of identity switches. These findings demonstrate the effectiveness of the COS strategy in mitigating target misidentification issues in environments with severe occlusion, enabling more reliable and accurate tracking in complex scenarios.

In summary, the incorporation of the aforementioned improvements in the YOLOv7 + Deep SORT framework for wildlife tracking applications yields practical benefits across multiple dimensions. These benefits are primarily reflected in the high precision, low energy consumption, and long-term continuous monitoring required for wildlife tracking. By enhancing detection and tracking accuracy, improving adaptability to small objects and occluded targets, and optimizing real-time performance and resource efficiency, this approach significantly enhances the reliability and data quality of field monitoring systems. It is particularly well-suited for multi-target tracking in wildlife conservation and research tasks.

However, the ablation experiments also revealed some unexpected results and patterns. For instance, while the COS enhances target detection in complex scenes, it can occasionally lead to increased false positives or missed detections during the tracking process in certain scenarios. This phenomenon is particularly pronounced when the target frequently enters and exits the field of view. Additionally, in scenes with high motion complexity, CMC may misinterpret target behavior as abnormal camera motion, resulting in incorrect adjustments to target motion features and causing abrupt jumps or loss of tracking trajectories.

In addition, ablation experiments have certain limitations. Firstly, there may be a degree of interdependence between modules, and the removal of a single module could impact the performance of other modules. This interdependence may prevent the experimental results from accurately reflecting the independent contribution of each module. Furthermore, the lack of universal evaluation metrics poses a challenge. Current metrics used for multi-target tracking, such as MOTA and IDF1, may not fully measure the individual contributions of each module. For example, while CMC enhances tracking stability, these metrics may not adequately capture the nuanced improvements brought about by compensation. The contributions of certain enhanced modules may only become apparent in specific scenarios, which are not effectively reflected by the existing evaluation metrics.

### 3.5. Actual tracking results

**Fig 11:**
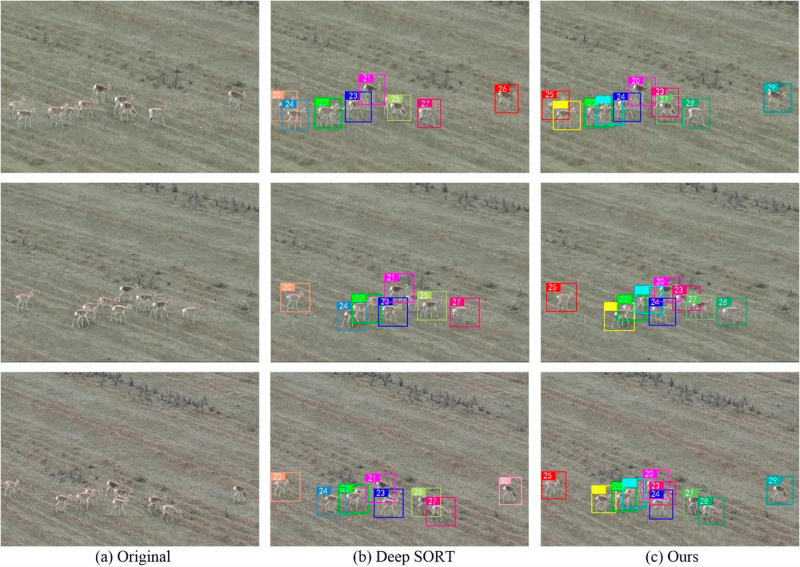
Comparison of tracking results. **(a)** Original image; **(b)** Deep SORT model; **(c)** Our method.

To demonstrate the capabilities of our tracking methodology, Fig 11 provides a side-by-side comparison of tracking visualizations under varied environmental conditions. The figure is divided into three sections: the first section presents the original scene images, the second section displays the tracking results using the traditional Deep SORT algorithm, and the third section illustrates the outcomes achieved with our advanced tracking method. Analysis of these images reveals that the conventional Deep SORT algorithm frequently fails to detect targets in scenarios involving occlusion, whereas our method effectively addresses these challenges, maintaining consistent detection accuracy. Furthermore, in scenarios where the target temporarily exits and later reenters the camera’s field of view, our method demonstrates a clear advantage by effectively retaining the target’s identification—a capability that the traditional Deep SORT algorithm often struggles to achieve. These comparative results highlight the enhanced ability of our method to handle occlusions and sustain continuous and accurate tracking of targets across diverse environmental conditions.

## 4. Discussion

With the continuous advancement of deep learning technology, significant strides have been made in the field of wildlife conservation. For instance, Schindler and Steinhage [41] developed a new MOT and Segmentation (MOTS) pipeline named SWIFT, which generates precise instance masks through a refinement process. This pipeline incorporates multiple filtering steps to effectively track and monitor wildlife populations. To further enhance the monitoring capabilities for wildlife, Cunha et al. [42] proposed a simple yet effective framework that employs multi-branch methods to improve the recognition of tail categories within the long-tail camera trap dataset, thus minimizing the performance loss in head categories. These algorithms enable a more accurate understanding of the distribution, abundance, and behavioral patterns of wild animals by enhancing detection accuracy. However, the monitoring range is limited, and it is restricted to video data that does not necessitate real-time analysis.

The current research focus is on utilizing drones combined with deep learning methods for real-time wildlife monitoring. Chen et al. [43] developed YOLO-SAG, an enhanced wildlife detection algorithm based on YOLOv8, designed to optimize the accuracy and speed of wildlife monitoring activities. This technology addresses challenges such as complex outdoor environments and diverse animal body types, which typically hinder detection accuracy. Roy et al. [44] introduced WilDect YOLO, an algorithm for the accurate automatic identification and localization of endangered animal populations. However, these methods still struggle to dynamically respond to movements within wild animal populations. Effective protection requires accurately capturing population dynamics [45,46], necessitating the development of precise and effective monitoring strategies.

High-altitude wildlife monitoring poses significant challenges due to the dynamic and complex scenarios encountered in target tracking. These challenges necessitate the development of an advanced model specifically tailored for environments characterized by both limited computational resources and intricate tracking conditions. The newly developed model addresses these challenges effectively. According to the data presented in Table 4, the metrics of our model, including MOTA, MOTP, and IDF1, register values of 69.3%, 78.9%, and 65.8%, respectively. These figures not only surpass those of other leading tracking models but also underscore the substantial advancements our model brings to tracking accuracy in high-altitude environments. The enhanced performance is particularly notable in scenarios involving occluded views and rapid movements of UAV-mounted cameras, which are prevalent in these regions. Such enhancements are crucial for the accurate monitoring and conservation of endangered wildlife, providing a reliable tool for researchers navigating the complexities of variable tracking landscapes.

The rapid advancements in deep learning technologies have significantly enhanced the capabilities of UAV in the field of wildlife research. Despite these technological strides, research employing UAV for wildlife monitoring remains relatively limited in scope. Common approaches in this domain predominantly focus on methodologies such as aerial counting [47] and object detection [48], facilitated by UAV. While these methods are effective at identifying diverse wildlife species from aerial perspectives, they face considerable challenges in maintaining persistent tracking of these animals, especially when they engage in frequent and rapid movements across their natural habitats.

In addressing the identified challenges in wildlife tracking, this study introduces a multi-target tracking framework based on the TBD paradigm, enabling sustained and effective monitoring of wildlife movements. The efficacy of this tracking system crucially hinges on the performance of its detection component. Given the computational constraints of our UAV-based platform, we meticulously balanced maintaining high accuracy with ensuring real-time processing capabilities, ultimately leading to the selection of the YOLOv7 detection model for this purpose. The capacity for feature representation in detection models is paramount, primarily evident in their ability to precisely identify and localize multiple targets within an image and provide accurate bounding boxes for these targets. To enhance this capacity, we integrated the GSConv convolution technique, known for capturing comprehensive global information, replacing the standard convolution in the YOLOv7 neck network. Additionally, the introduction of the lightweight Content Aware Feature Recombination (CARAFE) operator, which supplants the original nearest neighbor interpolation, significantly reduces the degradation of feature information during the upscaling process. According to the data presented in Table 4, the modifications to the detection model have resulted in a 4.7% increase in the mAP value, along with a reduction of 1.8 million in model parameters, affirming the substantial improvements made.

In this study, Deep SORT was meticulously selected as the baseline model for tracking targets. This model employs a sophisticated cascaded matching framework that not only considers the spatial positioning of the targets but also integrates their feature information across successive frames, ensuring robust tracking outcomes. Despite its effectiveness, challenges persist when deploying UAV for tracking fast-moving targets, primarily due to the camera’s susceptibility to vibrations that often disrupt tracking continuity. Moreover, severe occlusions frequently result in detection failures. To address these challenges, the study introduced a CMC module, designed to enhance the KF for superior motion estimation and tracking correlation under conditions of camera instability. The ablation study detailed in Table 4 highlights significant improvements; the integration of the CMC module increased the MOTA score from 63.8% to 66.2%, and the IDF1 score saw a rise of 2.1%. These results underscore the effectiveness of the CMC module in mitigating issues related to camera shake. Furthermore, to manage the complexities of tracking in environments with substantial occlusions, a COS was formulated. This strategy employs varying matching thresholds for detection boxes based on their confidence levels, offering a dynamic solution to effectively handle the challenges posed by complex occlusions. This approach has significantly diminished the instances of missed detections in scenarios characterized by dense occlusions, adapting seamlessly to the multifaceted conditions prevalent in wildlife monitoring and providing steadfast support for multi-target video tracking.

Despite the progress made in this research, inherent constraints still exist regarding the operational testing of our model under varying light conditions such as at dusk, dawn, and nighttime. In such scenarios, the tracking performance of this system may significantly decrease.

## 5. Conclusion

This article presents a novel multi-target tracking approach for UAV, utilizing YOLOv7 in combination with Deep SORT to monitor the Procapra przewalskii. Firstly, GSConv convolution and CARAFE up-sampling operator are integrated into the YOLOv7 neck, and GSConv is used to process features to improve the efficiency of convolution and obtain feature maps with efficient feature representation. Then, CARAFE is used for up-sampling to enhance details and ensure that the up-sampled feature map has sufficient resolution and delicacy to detect small targets and subtle structures. Through this combination, YOLOv7 can not only achieve faster inference speed, but also improve feature detail preservation, thereby improving detection accuracy. In addition, to ensure the stability of tracking, we have introduced CMC module (adjust video frames based on motion vectors to reduce blur caused by motion) and COS strategy (keep almost all detection boxes and classify them into high, medium, and low confidence categories based on different detection score thresholds) in Deep SORT. CMC can predict the tracking target through a stable Kalman filter, reduce the impact of global motion, and enable COS to work at a more realistic target position, thereby improving tracking accuracy and reducing the influence of target occlusion in complex environments. The superior performance of this proposed method has been validated through detailed ablation studies, comparative experiments, and the analysis of visual tracking results.

An extensive evaluation demonstrates that the system is highly effective in monitoring Procapra przewalskii. When compared to the original combination of YOLOv7 and Deep SORT, this enhanced system shows improvements in the MOTA, MOTP, and IDF1 metrics by 7.0%, 3.7%, and 5.8% respectively. These advancements confirm the superior performance of the proposed system. In conclusion, UAV equipped with this technology provide real-time surveillance capabilities that can deter poaching and contribute significantly to the preservation of the Procapra przewalskii, thereby playing a crucial role in maintaining ecological balance. The outcomes of this research are of substantial value and have considerable potential for widespread application.

Subsequent studies are planned to incorporate UAV equipped with thermal imaging technology to explore and refine tracking methodologies under the low-light conditions typical of early mornings and nights. This advancement is intended to facilitate continuous and effective monitoring of wildlife at all times of the day.

## Supporting information

S1 File Brief explanation of terms. (PDF)
