# Supplementary material for: A multi-target tracking method for UAV monitoring wildlife in Qinghai
Source: PLoS One. 2025 Apr 11;20(4):e0317286. doi: 10.1371/journal.pone.0317286 (PMC11990782; doi:10.1371/journal.pone.0317286)
Supplement: S1 File — (PDF) [file pone.0317286.s001.pdf]

Brief explanation of technical terms in this article:

Group-Selective Convolution (GSConv): A convolution operation that uses a grouping selection mechanism for feature extraction to enhance the computational efficiency and flexibility of convolutional networks. GSConv reduces computational complexity while maintaining detection accuracy by dividing features into multiple groups for selective convolution.

Content-Aware ReAssembly of FEatures(CARAFE): A content aware feature recombination module commonly used for feature upsampling. CARAFE intelligently generates upsampled feature maps by learning the relationships between local pixels, which preserves more details and feature information than traditional interpolation methods.

C**amera Motion Compensation (CMC):** The CMC module improves image stability by eliminating drone camera shake, thereby enhancing the multi-target tracking accuracy of the Deep SORT algorithm in dynamic environments.

Confidence Optimization Strategy (COS): The COS module enhances tracking robustness in situations of occlusion or visual interference by dynamically adjusting the system's confidence in the target.

Kalman filter (KF)： KF is a recursive algorithm that provides the optimal estimation of the dynamic system state by combining predicted and observed values, and is widely used in fields such as target tracking.

Extended High-Efficiency Layer Aggregation Network (E-ELAN): E-ELAN is a deep learning architecture that improves network performance and efficiency by fusing features at different levels. It enhances the expressive power of the network through layer aggregation, while adopting a lightweight design to reduce computational complexity and improve computational efficiency.

The FAST corner detection algorithmFAST: Quickly identify points of interest in the image by comparing the brightness of surrounding pixels.

BRIEF descriptor: By comparing local pixel pairs in binary, efficient and compact feature representations are provided.

The ORB approach： Combining FAST and BRIEF enhances rotational invariance and improves computational efficiency, making it suitable for real-time applications.

Re-ID： In DeepSORT, Re-ID is used to extract pedestrian appearance features through deep learning, and combined with Kalman filtering and Hungarian algorithm for multi-target tracking to achieve efficient and accurate target re recognition and tracking.

The Randomized Sampling Consistency Algorithm (RANSAC)： It is an iterative algorithm used to estimate model parameters from noisy or outlier data. In background motion estimation, RANSAC helps to calculate accurate affine transformation matrices through feature point matching, ensuring correct estimation of motion, especially exhibiting strong robustness when there is noise or incorrect matching in the data.
